# Codon Usage Analysis Reveals Distinct Evolutionary Patterns and Host Adaptation Strategies in Duck Hepatitis Virus 1 (DHV-1) Phylogroups

**DOI:** 10.3390/v16091380

**Published:** 2024-08-29

**Authors:** Yongwang Zhao, Xiaojian Su, Dongchang He

**Affiliations:** Department of Veterinary Medicine, Jiangsu Agri-Animal Husbandry Vocational College, Taizhou 225300, China

**Keywords:** duck hepatitis virus 1, phylogeny, codon usage bias, selection pressure, random genetic drift, adaptation

## Abstract

Duck hepatitis virus 1 (DHV-1) is a major threat to the global poultry industry, causing significant economic losses due to high mortality rates in young ducklings. To better understand the evolution and host adaptation strategies of DHV-1, we conducted a comprehensive codon usage analysis of DHV-1 genomes. Our phylogenetic analysis revealed three well-supported DHV-1 phylogroups (Ia, Ib, and II) with distinct genetic diversity patterns. Comparative analyses of the codon usage bias and dinucleotide abundance uncovered a strong preference for A/U-ended codons and a biased pattern of dinucleotide usage in the DHV-1 genome, with CG dinucleotides being extremely underrepresented. Effective number of codons (ENC) analysis indicated a low codon usage bias in the DHV-1 ORF sequences, suggesting adaptation to host codon usage preferences. PR2 bias, ENC plot, and neutrality analyses revealed that both mutation pressure and natural selection influence the codon usage patterns of DHV-1. Notably, the three DHV-1 phylogroups exhibited distinct evolutionary trends, with phylogroups Ia and Ib showing evidence of neutral evolution accompanied by selective pressure, while the phylogroup II evolution was primarily driven by random genetic drift. Comparative analysis of the codon usage indices (CAI, RCDI, and SiD) among the phylogroups highlighted significant differences between subgroups Ia and Ib, suggesting distinct evolutionary pressures or adaptations influencing their codon usage. These findings contribute to our understanding of DHV-1 evolution and host adaptation, with potential implications for the development of effective control measures and vaccines.

## 1. Introduction

Duck hepatitis virus (DHV), a member of the family *Picornaviridae*, causes highly contagious and fatal hepatitis that primarily affects young ducklings, leading to significant economic losses for the global poultry industry. Since its first identification in 1945 from outbreaks of duck viral hepatitis in Long Island, New York [1], DHV has been reported in various countries, with a high prevalence in major duck-farming regions such as China [2] and Vietnam [3]. The virus is classified into three distinct serotypes: DHV-1, DHV-2, and DHV-3 [4], each differing in genetic composition and geographical distribution [5,6]. Among these, DHV-1 is the most prevalent and virulent, causing mortality rates exceeding 80% in ducklings younger than three weeks [7]. The lack of cross-neutralization among these serotypes further complicates efforts to effectively manage and control outbreaks [4].

DHV-1 infection is characterized by a sudden onset and high mortality rates among ducklings within the first few weeks of life [8]. Clinical symptoms include lethargy, anorexia, and signs of hepatitis, such as enlarged and discolored livers [9]. The virus is transmitted through direct contact with infected ducks or contaminated environments. Age-related susceptibility is a critical factor, with young ducklings being particularly vulnerable to infection and severe disease outcomes [10]. Recent DHV-1 outbreaks worldwide have resulted in significant economic losses [11] and prompted the implementation of control measures, such as vaccination and enhanced surveillance.

Several studies have focused on genetic factors influencing DHV-1 virulence, aiming to identify key mutations or genomic regions associated with increased pathogenicity [12]. For instance, specific mutations in the VP0 and 2C proteins of DHV-1 have been linked to changes in the virus’s ability to infect and cause disease in ducklings [12]. Research on host–virus interactions has also provided insights into how DHV-1 evades the immune system or adapts to different host environments, contributing to its success as a pathogen [13]. Understanding the pathogenicity and epidemiology of DHV-1 is crucial for the poultry industry and has broader implications for virological research, such as deciphering cross-species transmission mechanisms and developing effective vaccines.

To develop effective control strategies and vaccines, it is crucial to understand the molecular mechanisms underlying DHV-1 pathogenicity and host adaptation. Codon usage bias, the preferential use of certain codons over others encoding the same amino acid, has been shown to influence viral replication, protein expression, and overall fitness [14]. Analyzing the codon usage patterns in viral genomes provides valuable insights into virus evolution and adaptation to host environments [15,16]. However, there is limited research exploring the direct link between the evolutionary pattern of DHV-1 viruses and the codon usage pattern and the adaptability of the virus in the host [17,18]. A comprehensive analysis of DHV-1 codon usage, considering different types and strains, is necessary to fill this knowledge gap and provide insights into the virus’s evolutionary dynamics and host adaptation strategies.

This study aims to conduct a thorough codon usage analysis of DHV-1 genomes, encompassing all the known types and strains. By employing bioinformatic tools and statistical methods, we will identify patterns of codon usage bias and determine the factors influencing those patterns, such as mutational pressure, natural selection, and host adaptation. Furthermore, we will explore the potential implications of codon usage bias for DHV-1 evolution, virulence, and host specificity. The findings of this study will provide a deeper understanding of the evolutionary dynamics and host adaptation strategies of DHV-1, potentially leading to novel strategies for disease control and prevention.

## 2. Materials and Methods

### 2.1. Data Collection and Sequence Retrieval

The dataset of duck hepatitis virus 1 (DHV-1) sequences was downloaded from the GenBank database of the National Center for Biotechnology Information (NCBI) as of April 2024. The dataset consisted of 100 complete DHV-1 genome sequences.

### 2.2. Recombination Detection and Phylogenetic Analysis

To identify potential recombination events in the coding DNA sequences (CDS) of the DHV-1 strains, the recombination detection program RDP4 (version 4.104) [19] was employed. The aligned sequences were subjected to recombination analysis using seven different recombination detection algorithms: RDP, GENECONV, Chimeara, MaxChi, BootScan, 3Seq, and SiSca. The default configurations were applied for each algorithm, and a stringent Bonferroni-corrected *p*-value cutoff of 10^−6^ was implemented throughout the analysis to minimize the false-positive results. To ensure the robustness of the recombination detection, only sequences supported by at least four different methods were considered to be recombinants. In cases where recombination signals were detected, the identified recombinant sequences were removed, and the left sequences were further analyzed using the same method mentioned above until no additional recombination events were identified. To further validate the results obtained from RDP4, sequence similarity plots were generated using the ggmsa package (version 1.10.0) [20] within the R software environment (version 4.4.0) [21].

The phylogenetic analysis was conducted using a two-step approach to infer the evolutionary relationships among the DHV-1 strains. First, the best-fitting nucleotide substitution model was determined using the Modelfinder [22] module in IQ-TREE [23] (version 1.6.12) software. The general time-reversible (GTR) model with gamma-distributed rate variation among sites (G) and a proportion of invariable sites (I) was identified as the optimal model (GTR + G+I). Next, the phylogenetic tree was reconstructed using both maximum likelihood (ML) methods to ensure robust and reliable evolutionary inferences. The ML analysis was performed using RAxML (version 8.2.12) [24], and the statistical support for individual branches was assessed using 1000 bootstrap replicates. The resulting phylogenetic trees were visualized and annotated using FigTree (version 1.4.4) (https://github.com/rambaut/figtree, accessed on 1 May 2023). The available host, date and location information for each sequence was retrieved from the Nucleotide database and the literature. This information was then included next to the corresponding branch on the phylogenetic tree. The tree was rooted using the midpoint rooting method, which places the root at the midpoint of the longest branch.

### 2.3. Pairwise Genetic Distance Analysis

For a comprehensive comparison of the evolutionary distances and diversity patterns among the DHV-1 phylogroups, the pairwise genetic distances among the three identified phylogroups were estimated using the DIVEIN software [25]. The general time-reversible (GTR) model of nucleotide substitution with a gamma distribution (Γ) of rate variation across sites, selected as the best-fit model by ModelFinder [22], was employed for the distance calculations. To assess the statistical significance of the differences in intra-individual genetic sequence diversity between the phylogroups, a two-sample t-test was performed within the DIVEIN platform [25].

To address the potential impact of unequal sample sizes on the genetic diversity estimates, we employed a subsampling approach. For the subsampling analysis, we randomly selected 15 sequences (the smallest sample size among the phylogroups) from each phylogroup and repeated the genetic diversity analyses using these subsampled datasets. This process was repeated 100 times to generate a distribution of diversity estimates for each phylogroup, accounting for potential sampling biases.

### 2.4. Compositional and Principal Component Analysis

A detailed compositional analysis of the complete CDS of the DHV-1 strains was conducted, excluding the five non-synonymous codons (AUG, UGG, and the three termination codons). The frequencies of individual mononucleotides (A, C, U, and G) and the GC contents at the first (GC1s), second (GC2s), and third (GC3s) codon positions, as well as the mean GC content of the first and second positions (GC12s), were calculated using the seqinr package (version 3.6-1) [26] in R (version 4.4.0) [21].

### 2.5. Relative Synonymous Codon Usage Analysis

Relative synonymous codon usage (RSCU) analysis was performed to assess the codon usage bias in the DHV-1 coding sequences, independent of the influence of the nucleotide composition and sequence length [27]. The RSCU value for each codon was calculated using the following equation [28]:

(1) $$RSCU=\frac{g_{ij}}{\sum_{j=1}^{ni}g_{ij}}n_{i}$$

In the equation, *g_ij_* represents the observed count of the *i_th_* codon for the *j_th_* amino acid, and *n_i_* denotes the number of synonymous codons for the *j_th_* amino acid. The RSCU values were computed using the seqinr package (version 3.6-1) [26] in R (version 4.4.0) [21]. An RSCU value of 1.0 indicates no codon usage bias, while values greater than 1.0 and less than 1.0 represent positive and negative bias, respectively. Codons with RSCU values exceeding 1.6 were considered “over-represented”, whereas those with values below 0.6 were deemed “under-represented” [29]. The contribution values of each codon to the overall codon usage bias were calculated based on the first two principal components. The top 10 codons with the highest contribution values were selected for display in the PCA biplot to highlight the most influential factors driving the differences in codon usage patterns among the DHV-1 phylogroups.

### 2.6. Principal Component Analysis

To explore the major trends and patterns in codon usage across the DHV-1 strains, a principal component analysis (PCA) was conducted using the RSCU values as input variables. PCA is a widely used unsupervised linear transformation technique for feature extraction and dimensionality reduction [30]. A matrix containing the RSCU values for the 59 sense codons was constructed for each DHV-1 sequence. The PCA was performed using the factoextra package (version 1.0.6) [31] in R (version 4.4.0) [21], which transformed the high-dimensional RSCU data into a reduced set of orthogonal principal components (PCs). The resulting PCs were visualized to identify the main sources of variation in codon usage among the DHV-1 strains and to detect potential evolutionary or functional trends.

### 2.7. Relative Dinucleotide Abundance Analysis

To further investigate the compositional patterns and potential biases in the DHV-1 genome, the relative abundances of the 16 dinucleotides were calculated using the following formula [32]:

(2) $$P_{xy}=\frac{f_{xy}}{f_{x}f_{y}}$$

where *f_x_* and *f_y_* represent the frequencies of nucleotides X and Y, respectively, *f_xy_* denotes the observed frequency of the dinucleotide XY, and *P_xy_* represents the relative abundance of the dinucleotide XY. Dinucleotides with *P_xy_* values greater than 1.23 were considered over-represented, while those with values less than 0.78 were deemed under-represented. The extent of the dinucleotide representation was further categorized as follows [33]: extremely over-represented (*P_xy_* ≥ 1.50), very over-represented (1.30 ≤ *P_xy_* < 1.50), significantly over-represented (1.23 ≤ *P_xy_* < 1.30), marginally over-represented (1.20 ≤ *P_xy_* < 1.23), extremely under-represented (*P_xy_* ≤ 0.50), very under-represented (0.50 < *P_xy_* ≤ 0.70), significantly under-represented (0.70 < *P_xy_* ≤ 0.78), and marginally under-represented (0.78 < *P_xy_* ≤ 0.81).

### 2.8. Effective Number of Codons Analysis

The effective number of codons (ENC) analysis was performed to quantify the extent of codon usage bias in the DHV-1 coding sequences, independent of the gene length and amino acid composition [34]. ENC values range from 20 to 61, with lower values indicating higher codon usage bias and higher values suggesting more even codon usage [34,35]. An ENC value less than or equal to 45 is considered indicative of strong codon usage bias. The ENC values for each DHV-1 sequence were calculated using the following formula [34]:

(3) $$\mathrm{ENC}=2+\frac{9}{{\bar{F}}_{2}}+\frac{1}{{\bar{F}}_{3}}+\frac{5}{{\bar{F}}_{4}}+\frac{3}{{\bar{F}}_{6}}$$

where ${\bar{F}}_{i}$ (*i* = 2, 3, 4, 6) represents the average $\bar{F}$ value for the *i*-fold degenerate amino acid family. The ${\bar{F}}_{i}$ values were computed using the following equation [34]:

(4) $${\bar{F}}_{i}=\frac{n\sum_{j=1}^{i}{\left(\frac{n_{j}}{n}\right)}^{2}-1}{n-1}$$

where *n* denotes the total number of observed codons for a given amino acid, and *n_j_* represents the observed count of the *j_th_* codon for that amino acid. The ENC values were calculated using the cordon package (version 1.4.0) [36] in R (version 4.4.0) [21].

### 2.9. ENC Plot Analysis

To investigate the factors influencing codon usage bias in the DHV-1 genome, an ENC plot analysis was conducted. The ENC plot displays the relationship between the GC content at the third codon position (GC3) and the ENC values [34]. If the observed ENC values lie on or near the expected ENC curve, it suggests that the codon usage is primarily influenced by mutation pressure. Deviations of the observed ENC values below the expected curve indicate the influence of additional factors, such as natural selection, on the codon usage bias. The expected ENC values were calculated using the following formula:

(5) $${ENC}_{expected}=2+s+\frac{29}{s^{2}+{\left(1-s\right)}^{2}}$$

where *s* represents the frequency of G or C at the third position of synonymous codons.

### 2.10. Parity Rule 2 Analysis

Parity rule 2 (PR2) analysis was employed to assess the relative contributions of natural selection and mutation pressure to the codon usage bias in the DHV-1 genome. In the PR2 plot, the ordinate represents the frequency of A at the third codon position [*A*3/(*A*3 + *U*3)], while the abscissa represents the frequency of G at the third codon position [*G*3/(*G*3 + *C*3)]. The origin point (0.5, 0.5) indicates equal usage of A and T (A = T) and equal usage of G and C (G = C). Deviations from the origin point suggest the influence of natural selection and mutation pressure on the codon usage.

### 2.11. Neutrality Analysis

Neutrality analysis was conducted to investigate the relative contributions of natural selection and mutation pressure to the codon usage bias in the DHV-1 genome. This analysis involves examining the relationship between the GC content at the third codon position (GC3s) and the average GC content at the first and second codon positions (GC12s) [37]. A linear regression analysis was performed using R (version 4.4.0) [21] to assess the strength and significance of this relationship. If the regression coefficient (slope) is close to 1 and statistically significant, mutation pressure is considered the primary force shaping the codon usage. Conversely, a slope closer to 0 indicates a diminishing effect of mutation pressure, and a slope of 0 suggests that the codon usage bias is entirely determined by natural selection [37].

### 2.12. Codon Adaptation Index Analysis

The codon adaptation index (CAI) was calculated to evaluate the adaptiveness of DHV-1 genes to the codon usage patterns of highly expressed genes in the host species (*Anas platyrhynchos domesticus*) [38]. CAI values range from 0 to 1, with higher values indicating greater adaptiveness and potential for efficient expression. The CAI values for all the DHV-1 genes were computed using the CAIcal tool [39]. The reference dataset of synonymous codon usage patterns for *Anas platyrhynchos domesticus* was obtained from the Codon and Codon Pair Usage Tables (CoCoPUTs) database [40] (updated in September 2021).

### 2.13. Relative Codon Deoptimization Index Analysis

The relative codon deoptimization index (RCDI) was employed to assess the similarity in codon usage between the DHV-1 genes and the host reference genome. RCDI values close to 1 suggest a high degree of similarity between the codon usage of the pathogen and the host, potentially leading to higher translation rates [41]. The RCDI values were calculated using the CAIcal tool [39].

### 2.14. Similarity Index Analysis

The similarity index (SiD) was used to quantify the influence of host codon usage on the codon usage of the DHV-1 genome. The SiD values were calculated using the following equations [41]:

(6) $$R\left(A,B\right)=\frac{\sum_{i=1}^{59}a_{i}\times b_{i}}{\sqrt{\sum_{i=1}^{59}a_{i}^{2}\times \sum_{i=1}^{59}b_{i}^{2}}}$$

(7) $$D(A,B)=\frac{1-R(A,B)}{2}$$

where *a_i_* represents the RSCU value for a specific synonymous codon in the DHV-1 coding sequence, and *b_i_* is the corresponding RSCU value for the same codon in the host genome. *D*(*A*, *B*) quantifies the overall influence of host codon usage on the codon usage of the pathogen. Higher SiD values indicate a greater influence of the host on the pathogen’s codon usage.

### 2.15. Statistical Analysis

Due to the non-normal distribution and unequal variances of the CAI, SiD, and RCDI values across the DHV-1 phylogroups, non-parametric statistical tests were employed. The Kruskal–Wallis test, followed by the Bonferroni-corrected Dunn’s multiple comparison test, was used to assess the statistical significance of the differences in CAI, SiD, and RCDI values among the phylogroups. The dunn.test package (version 1.3.5) in R (version 4.4.0) [21] was utilized for these analyses.

## 3. Results

### 3.1. Recombination and Phylogenetic Analysis

A total of 117 DHV-1 complete CDSs were obtained from GenBank in April 2024. Following the removal of incomplete sequences, unverifiable sequences, and sequences belonging to the vaccine strain, a total of 100 sequences remained. To avoid the potential effects of recombination on the topology of the phylogenetic tree, the DHV-1 CDSs were analyzed using the recombination detecting program RDP4 (version 4.101) [19]. Two sequences of DHV-1 strains were found to have potential recombination signals (Figure S1). After removing the recombinant DHV-1 CDSs, the remaining 98 sequences were used for further analysis.

**Figure 1:**
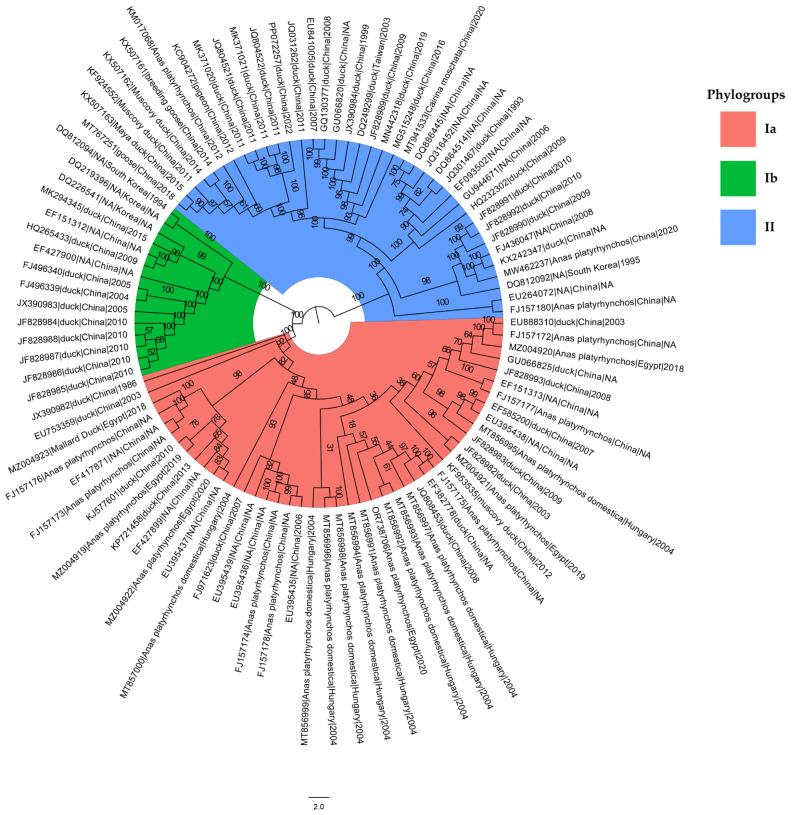
Maximum likelihood tree of the complete coding sequences (CDS) of DHV-1. Bootstrap support values computed by RAxML are indicated on the nodes of the tree. The scale bar at the bottom of the figure corresponds to 2 nucleotide substitutions per site. The colored sectors represent three phylogroups of DHV-1. The tree is rooted using the midpoint rooting method. The host, location and date information for each sequence is provided. N’A’ (not available) indicates that the information is not available for the particular sequence.

To investigate the evolutionary relationships among the DHV-1 strains, maximum likelihood (ML) phylogenetic trees were reconstructed. The analysis revealed that all the known DHV-1 strains could be classified into three well-supported phylogroups: Ia, Ib, and II (Figure 1). This phylogenetic grouping provided a framework for further comparative analyses of genetic diversity and evolutionary patterns within and between the identified DHV-1 phylogroups. The analysis of the pairwise genetic distances highlighted substantial differentiation between the major phylogroups I and II (mean distance: 0.07159 ± 0.00034) and considerable divergence within phylogroup I between subgroups Ia and Ib (mean distance: 0.06046 ± 0.00066). Phylogroup II displayed great genetic diversity (mean genetic sequence diversity: 0.05687 ± 0.00093), while phylogroup I exhibited less diversity (0.03015 ± 0.00066). Within phylogroup I, subgroup Ia showed a lower diversity 0.008819 ± 0.00024), contrasting with the higher diversity in subgroup Ib (0.03650 ± 0.00210). Statistical tests confirmed the highly significant differences in genetic diversity between subgroups Ia and Ib (*Z* test *p* = 3.19 × 10^−8^; *T* test *p* = 4.27 × 10^−8^), and between phylogroups I and II (*Z* test *p* = 1.64 × 10^−6^; *T* test *p* = 1.04 × 10^−5^). These results suggest distinct evolutionary processes shaping the genetic composition of the DHV-1 phylogroups, potentially influenced by factors such as host adaptation or selective pressures.

Subsampling analysis revealed that the overall patterns of genetic diversity remained consistent with our original findings. The relative levels of genetic diversity within each phylogroup were maintained, with phylogroup II exhibiting the highest diversity (median = 0.0557, IQR (Interquartile Range) = 0.00437), followed by phylogroup Ib (median = 0.0355, IQR = 0.000276) and phylogroup Ia (median = 0.00826, IQR = 0.00324) (Figure S2). A Kruskal–Wallis test confirmed the significant differences among the phylogroups (*χ*^2^ = 265.78, *p* < 2.2 × 10^−16^). Dunn’s test with the Bonferroni correction further supported these results, showing significant differences between each pair of phylogroups (alpha = 0.05, reject Ho if *p* ≤ alpha/2) (Figure S2).

### 3.2. A Bias towards A and U in DHV-1 Coding Sequences

The mean composition of nucleotide A (0.292 ± 0.002) was the highest among the four nucleotides in the DHV-1 coding sequences, followed by U (0.281 ± 0.003), G (0.226 ± 0.001), and C (0.200 ± 0.003) (Table 1 and Table S1). The nucleotide composition at the third positions of the synonymous codons (N3) exhibited a different pattern, with U3 (0.350 ± 0.009) being the most abundant, followed by A3 (0.266 ± 0.004), G3 (0.202 ± 0.003), and C3 (0.182 ± 0.008), indicating the A/U-end codons were enriched in the DHV-1 coding sequences. The mean contents of GC and AT were 0.426 ± 0.003 and 0.574 ± 0.003, respectively. Among the GC contents at different codon positions, GC1 (0.492 ± 0.002) was the highest, followed by GC12 (0.448 ± 0.001), GC2 (0.403 ± 0.001), and GC3 (0.384 ± 0.009). The effective number of codons (ENC) values for the DHV-1 strains were 51.387 ± 0.233, 50.595 ± 0.872, and 51.175 ± 0.712 for phylogroups Ia, Ib, and II, respectively. These ENC values, falling within the range of 50–61, suggest a low codon usage bias in the DHV-1 ORF sequences.

### 3.3. Unique Relative Synonymous Codon Usage of DHV-1

**Figure 2:**
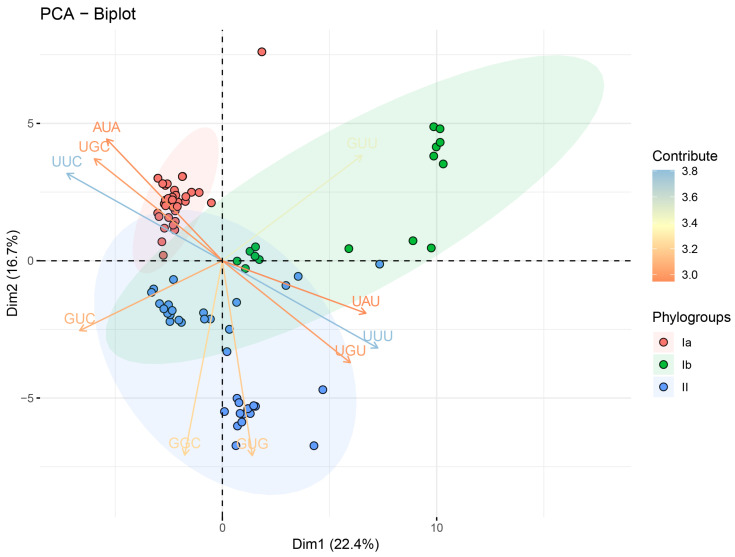
Principal component analysis (PCA) based on the relative synonymous codon usage (RSCU) values of the DHV-1 complete coding sequences. The first and second PCs collectively explained 39.1% of the total RSCU variation. Significant separation in codon usage bias among the three phylogroups of DHV-1 is present. Phylogroups Ia, Ib, and II are represented in orange, green, and blue, respectively. The top 10 codons with the highest contribution values to the overall codon usage bias are displayed in the biplot, as these codons are the most influential in driving the differences among the phylogroups.

One of the most striking features observed in the RSCU analysis was the preferential use of A/U-ended codons. Among the 18 preferred codons (RSCU > 1) shared by all three phylogroups, 9 were U-ended (AAU [Asn], GAU [Asp], UGU [Cys], CAU [His], AUU [Ile], UUU [Phe], UCU [Ser], UAU [Tyr], and GUU [Val]), 8 were A-ended (GCA [Ala], AGA [Arg], CAA [Gln], GAA [Glu], GGA [Gly], AAA [Lys], CCA [Pro], and ACA [Thr]), and only 1 was G-ended (UUG [Leu]) (Table S2). A small proportion of codons (11.86%, 7 out of 59) were over-represented (RSCU > 1.6) across all the phylogroups, including AGA [Arg], AUU [Ile], UUG [Leu], CCA [Pro], UCA [Ser], UCU [Ser], and ACA [Thr]. In contrast, a higher percentage of codons (20.34%, 12 out of 59) were under-represented (RSCU < 0.6), including GCG [Ala], CGU [Arg], AAC [Asn], UGC [Cys], CUA [Leu], UUC [Phe], CCG [Pro], AGC [Ser], UCG [Ser], ACG [Thr], UAC [Tyr], and GUA [Val]. Notably, four codons exhibited extremely low usage frequencies (RSCU values close to or less than 0.1): GCG [Ala] (0.086 ± 0.04), CCG [Pro] (0.06 ± 0.035), UCG [Ser] (0.108 ± 0.039), and ACG [Thr] (0.083 ± 0.035). Although the overall RSCU patterns were similar among the three DHV-1 phylogroups, some variations were observed in the usage of under-represented codons. Using the RSCU values as descriptor variables, an unsupervised classification method, principal component analysis (PCA), was performed to explore the codon usage features and evolutionary trends of DHV-1. The first and second principal components accounted for 22.4% and 16.7% of the total RSCU variations, respectively (Figure 2, Figure S3 and Figure S4). The PCA biplot (Figure 2) shows the top 10 codons that contribute the most to the overall codon usage bias, as determined by their contribution values. These codons were selected to highlight the most influential factors driving the differences in codon usage patterns among the DHV-1 phylogroups. The PCA plot revealed three distinctly separate groups, consistent with the phylogenetic relationships of the DHV-1 strains identified by the maximum likelihood (ML) analysis.

### 3.4. Relative Dinucleotide Abundance of DHV-1

**Figure 3:**
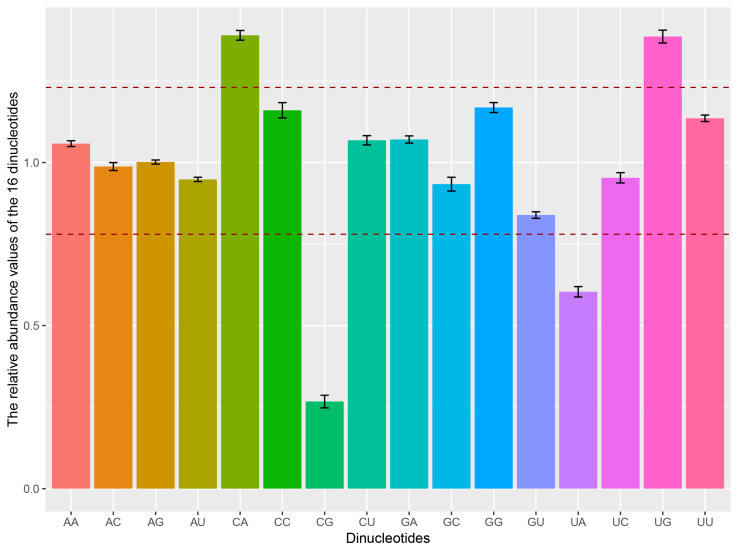
Relative dinucleotide abundance of the complete CDS of DHV-1. The dashed lines above and below represent 1.23 and 0.78, respectively.

Considering the relative abundance of dinucleotides affecting the pattern of codon usage in RNA viruses, we calculated the relative abundance of 16 dinucleotides for the DHV-1 ORF sequences. The relative abundance values of dinucleotides AC (0.988 ± 0.012) and AG (1.001 ± 0.006) were close to the theoretical value (equal to 1.0), while the other dinucleotides exhibited varying usage frequencies (Figure 3 and Table S3). Dinucleotides CA (*P_xy_* = 1.39 ± 0.015) and UG (*P_xy_* = 1.386 ± 0.02) were over-represented (*P_xy_* ≥ 1.23), while dinucleotide UA (*P_xy_* = 0.604 ± 0.016) was under-represented (*P_xy_* ≤ 0.78). Dinucleotide CG (*P_xy_* = 0.267 ± 0.019) was extremely under-represented (*P_xy_* ≤ 0.50) compared to the other dinucleotides, which could be attributed to the suppressive effect of CG dinucleotides on viral mRNA translation efficiency and stability. Several dinucleotides, including AA, CA, CC, CU, GA, GG, UG, and UU, exhibited usage frequencies greater than 1, while others, such as AU, UA, GU, and UC, had frequencies below 1, suggesting a selective preference for different dinucleotides in the DHV-1 genome. These findings indicate a biased pattern of dinucleotide usage in the DHV-1 genome.

### 3.5. The Effect of Mutation Pressure and Natural Selection on Codon Usage Bias

**Figure 4:**
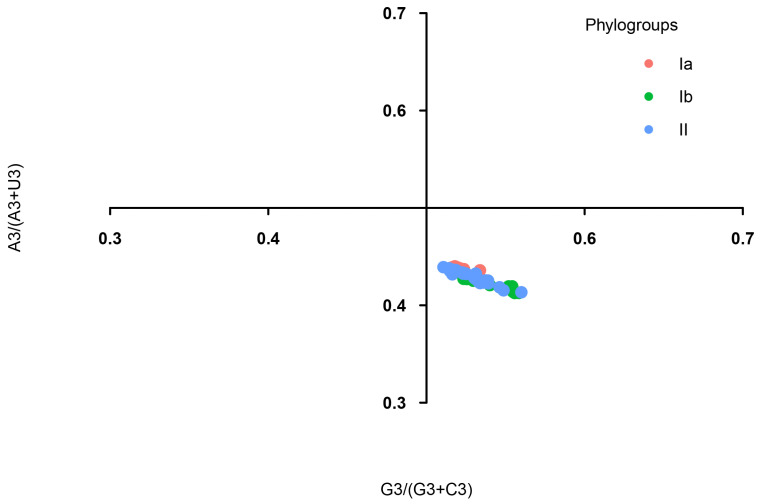
Parity rule 2 (PR2) plot analysis of the DHV-1 complete CDS. Phylogroups Ia, Ib, and II are represented in orange, green, and blue, respectively. The horizontal and vertical axes represent G3/(G3 + C3) and A3/(A3 + U3), respectively.

To explore the factors that influence the codon usage pattern, PR2 bias analysis, ENC plot analysis, and neutrality analyses of the different genotypes were employed. In the PR2 analysis, it was observed that all the data points fell within the fourth quadrant (Figure 4), indicating a preference for G over C and a preference for U over A at the third position of the codons. Specifically, this implies that among codons ending with either G or C, those terminating with G were used more frequently than those ending with C. Similarly, among codons ending with either A or U, those concluding with U exhibited a higher usage frequency compared to those ending with A. These findings suggest a distinct trend in the relative usage of G compared to C and U compared to A, respectively, at the third codon position in the DHV-1 genome. This tendency may not be merely random or solely determined by the GC content but rather reflects a complex interplay of various biological selection pressures and functional requirements.

**Figure 5:**
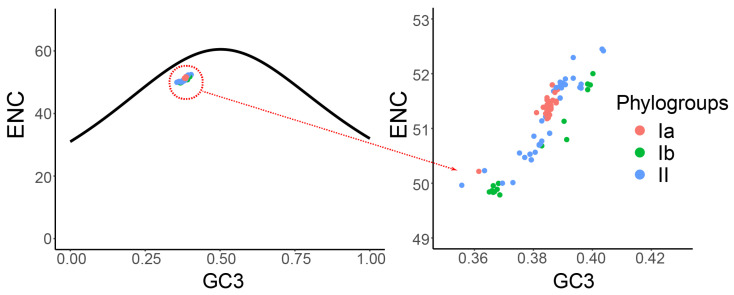
Effective number of codons (ENC) plot analysis of the DHV-1 complete CDS. The relationships between the ENC values and GC contents at the third codon position (GC3s) of synonymous codons are represented. The expected curve represents the expected ENC values for all the GC3 compositions. Phylogroups Ia, Ib, and II are represented in orange, green, and blue, respectively.

In the ENC plot analysis, all the data points were found to lie below the theoretical curve (Figure 5), indicating that codon usage bias in this gene or genome is influenced by factors beyond pure nucleotide composition. This observation suggests the presence of additional codon usage preferences that may be driven by specific biological selection pressures or adaptative requirements.

**Figure 6:**
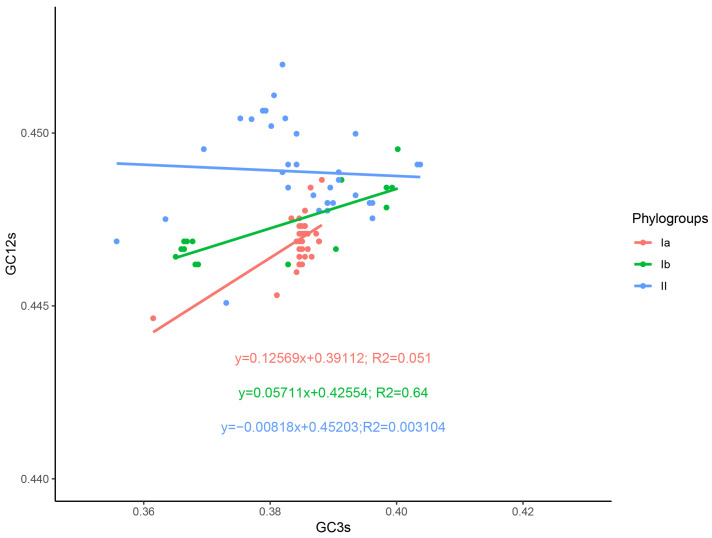
Neutrality analysis of the DHV-1 complete CDS. The correlation between GC12 and GC3 is represented. Phylogroups Ia, Ib, and II are represented in orange, green, and blue, respectively.

The neutrality analysis of these three evolutionary branches revealed distinct evolutionary trends. The results for phylogroups Ia and Ib indicated a positive correlation between the increase in the transition/transversion ratio and the increase in the third-position nucleotide diversity (Figure 6). Specifically, phylogroup Ia (R^2^ = 0.33, *p* = 3.64 × 10^−5^) exhibited the possibility of neutral evolution accompanied by slight selective pressure. Phylogroup Ib (R^2^ = 0.64, *p* = 0.000365) showed a manifestation of neutral evolution coexisting with a certain degree of selective pressure, although this pressure is not strong. The results of these two branches highlighted the differences between them. In contrast, the analysis of phylogroup II (R^2^ = 0.003104, *p* = 0.7397) demonstrated nearly no correlation between the transition/transversion ratio and the third-position nucleotide diversity. This suggests that the evolution of this branch is more likely to be entirely driven by random genetic drift, which is a typical characteristic of neutral evolution. Taken together, these findings reveal the potential existence of different genetic evolutionary mechanisms across the evolutionary branches. Each phylogroup may be influenced by distinct selective pressures and genetic drift, leading to the observed variation in their evolutionary patterns. This underscores the complexity and diversity of the evolutionary process.

### 3.6. Comparison between the Codon Pattern of Phylogroups toward Its Host

**Figure 7:**
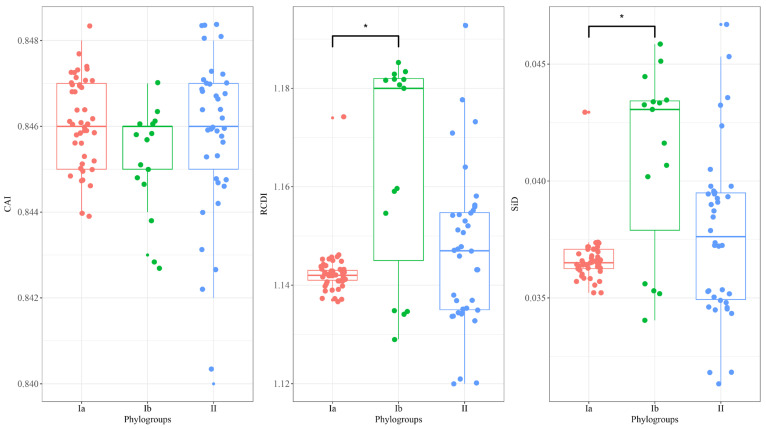
Analysis of DHV-1 adaptation to the host. Asterisks indicate significant differences at the 5% level. Phylogroups Ia, Ib, and II are represented in orange, green, and blue, respectively. Asterisk (*) indicates *p* < 0.05.

The Kruskal–Wallis rank sum test for the CAI values is not significant (*p* > 0.05) among the three phylogroups (Figure 7), suggesting that the codon adaptation index does not significantly differ among the three phylogroups of DHV-1. The lack of significant differences in the CAI values indicates that the overall codon adaptation to the host’s preferred codons may be similar across the phylogroups. In contrast, the Kruskal–Wallis rank sum test for the RCDI values among the phylogroups showed a significant difference (*p* = 0.009369) among the phylogroups (Figure 7). The post hoc Dunn’s test with the Bonferroni correction (alpha = 0.05, reject Ho if *p* ≤ alpha/2) revealed a significant difference between phylogroups Ia and Ib (*p* = 0.0034), while the differences between Ia and II (*p* = 0.4517) and between Ib and II (*p* = 0.0375) were not significant after the Bonferroni adjustment. These findings imply that the relative codon deoptimization index varies significantly among the phylogroups, with a notable difference between Ia and Ib. Similarly, the Kruskal–Wallis rank sum test for the SiD values is significant (*p* = 0.00667) among the phylogroups (Figure 7). The post hoc Dunn’s test with the Bonferroni correction (alpha = 0.05, reject Ho if *p* ≤ alpha/2) indicated a significant difference between phylogroups Ia and Ib (*p* = 0.0023), while the differences between Ia and II (*p* = 0.3693) and between Ib and II (*p* = 0.0361) were not significant after the Bonferroni adjustment. These results suggest that the similarity index also differs significantly among the phylogroups, with a prominent difference between Ia and Ib. The observed differences in the RCDI and SiD values among the phylogroups, particularly between Ia and Ib, might indicate distinct evolutionary pressures or adaptations influencing the codon usage in these groups.

## 4. Discussion

Duck hepatitis virus 1 (DHV-1) poses a significant threat to the global poultry industry, causing substantial economic losses due to its high mortality rates in young ducklings [1,2]. Understanding the evolution and host adaptation strategies of DHV-1 is crucial for developing effective control measures and vaccines. In this study, we conducted a comprehensive codon usage analysis of DHV-1 genomes, encompassing all the known types and strains, to gain insights into the virus’s evolutionary patterns and host adaptation mechanisms. Our main findings include the identification of three well-supported DHV-1 phylogroups with distinct genetic diversity patterns, a strong preference for A/U-ended codons in DHV-1 coding sequences, and the influence of both mutation pressure and natural selection in shaping the codon usage patterns of DHV-1.

The significant differences in genetic diversity between subgroups Ia and Ib, and between phylogroups I and II, suggest that different evolutionary processes, such as neutral evolution, natural selection, and genetic drift, may be shaping the genetic composition of these groups. While phylogroups Ia and Ib showed evidence of neutral evolution accompanied by weak selective pressure, phylogroup II appeared to be primarily influenced by random genetic drift. The distinct evolutionary patterns observed in each phylogroup underscore the complex evolutionary dynamics of DHV-1, with each group potentially experiencing a unique combination of evolutionary forces. The higher genetic diversity observed in phylogroup II compared to phylogroup I might suggest adaptation to a wider range of host species or environmental conditions, but further research is needed to confirm this hypothesis. These observed differences could be influenced by factors such as host adaptation or selective pressures, highlighting the need for further research to elucidate the underlying mechanisms driving these evolutionary patterns.

One limitation of our study is the unequal sample sizes across the DHV-1 phylogroups, which could potentially affect the genetic diversity estimates, particularly if the sequences within a given group were more closely related in space or time. The subsampling analysis, which involved randomly selecting an equal number of sequences from each phylogroup and repeating the genetic diversity analyses 100 times, revealed that the overall patterns of genetic diversity remained consistent with our original findings. However, we acknowledge that future studies should aim to validate our findings using larger and more balanced datasets to ensure the robustness of these conclusions.

Our finding about the preferential use of A/U-ended codons in the DHV-1 coding sequences is consistent with a previous study [18]. While their study provided insights into the codon usage patterns of DHV-1, our study expands upon their findings by incorporating a larger dataset of DHV-1 genomes, encompassing all the known strains. This comprehensive approach allows us to identify distinct evolutionary patterns and host adaptation strategies among the three main DHV-1 phylogroups (Ia, Ib, and II). The low codon usage bias (ENC values between 50–61) observed in the DHV-1 ORF sequences suggests that the virus may have evolved to adapt to its host’s codon usage preferences, potentially enhancing its ability to infect and replicate in host cells.

Furthermore, the relative dinucleotide abundance analysis revealed a biased pattern of dinucleotide usage in the DHV-1 genome, with CG dinucleotides being extremely under-represented. This finding is consistent with the observed suppression of CG dinucleotides in many RNA viruses [42], which is thought to enhance the viral mRNA translation efficiency and stability [32]. The biased dinucleotide usage patterns in DHV-1 may contribute to its evolutionary dynamics and host adaptation strategies, warranting further investigation into the functional consequences of these patterns for viral fitness and pathogenicity.

Our study employed PR2 bias analysis, ENC plot analysis, and neutrality analyses to investigate the factors influencing codon usage bias in DHV-1. The PR2 bias analysis revealed a preference for G and U at the third position of the codons, suggesting that the codon usage patterns in DHV-1 are shaped by a complex interplay of biological selection pressures and functional requirements. The ENC plot analysis further indicated that the codon usage bias in DHV-1 is influenced by factors beyond pure nucleotide composition, such as specific biological selection pressures or adaptive requirements. The neutrality analysis uncovered distinct evolutionary trends among the three DHV-1 phylogroups. The positive correlation between the transition/transversion ratio and the third-position nucleotide diversity in phylogroups Ia and Ib suggests the presence of neutral evolution accompanied by selective pressure, with a more pronounced effect in phylogroup Ib. In contrast, the lack of correlation in phylogroup II implies that its evolution is primarily driven by random genetic drift. These findings highlight the potential existence of different genetic evolutionary mechanisms across the DHV-1 phylogroups, with each group influenced by a unique combination of selective pressures and genetic drift. The comparison of the codon usage indices (CAI, RCDI, and SiD) among the phylogroups revealed significant differences between subgroups Ia and Ib, despite similar overall codon adaptation to the host’s preferred codons. These differences suggest that distinct evolutionary pressures or adaptations may be influencing the codon usage in these subgroups, potentially impacting virus–host interactions and pathogenicity.

The relationship between codon usage bias and viral pathogenicity is an important aspect to consider when studying the evolution and adaptation of viruses. In the case of DHV-1, our analysis revealed distinct patterns of codon usage bias among the different phylogroups, which may have implications for the virus’s pathogenicity and virulence. Previous studies have suggested that codon usage bias can influence viral replication, protein expression, and overall fitness, which are key factors determining the pathogenicity of a virus [14,15]. Viruses with codon usage patterns better adapted to their host’s translational machinery may have increased replication efficiency and protein production, potentially leading to enhanced virulence [16]. In our study, we observed a strong preference for A/U-ended codons in the DHV-1 genome, which may reflect an adaptation to the codon usage of the host species. This adaptation could enable the virus to exploit the host’s translational resources more efficiently, resulting in higher viral replication rates and, consequently, increased pathogenicity. Furthermore, the distinct codon usage patterns observed among the DHV-1 phylogroups may indicate different levels of adaptation to the host’s codon usage preferences. The variations in codon usage bias between phylogroups Ia, Ib, and II could potentially contribute to differences in their pathogenicity and virulence. For example, phylogroups with codon usage patterns better optimized for the host’s translational machinery may exhibit higher replication rates and increased virulence compared to other phylogroups.

To further investigate the relationship between codon usage bias and pathogenicity in DHV-1, future studies could focus on comparing the codon usage patterns of strains with varying levels of virulence. By analyzing the codon usage of highly pathogenic strains and comparing them to less virulent strains, researchers may identify specific codon usage signatures associated with increased pathogenicity. Additionally, in vitro and in vivo experiments could be conducted to assess the impact of codon optimization on viral replication, protein expression, and disease severity in DHV-1. Understanding the link between codon usage bias and pathogenicity in DHV-1 has important implications for the development of antiviral strategies and vaccines. By identifying the codon usage patterns associated with increased virulence, researchers can design targeted therapies that disrupt the virus’s ability to exploit the host’s translational resources. Moreover, this knowledge can guide the development of live-attenuated vaccines by modifying the codon usage of vaccine strains to reduce their pathogenicity while maintaining immunogenicity. Our findings on the distinct codon usage patterns among DHV-1 phylogroups provide a foundation for further investigating the relationship between codon usage bias and pathogenicity in this virus. Future studies exploring this link could offer valuable insights into the evolution and adaptation of DHV-1, ultimately contributing to the development of effective control measures and vaccines against this economically important poultry pathogen.

In conclusion, our comprehensive codon usage analysis of DHV-1 genomes has revealed distinct evolutionary patterns and host adaptation strategies among the virus’s three main phylogroups (Ia, Ib, and II). The identification of these phylogroups, each with unique genetic diversity patterns and codon usage biases, highlights the importance of considering evolutionary factors in understanding DHV-1 epidemiology and developing control strategies. The preferential use of A/U-ended codons, the biased dinucleotide usage, and the influence of both mutation pressure and natural selection on codon usage bias further emphasize the complex evolutionary dynamics of DHV-1. These findings contribute to our understanding of DHV-1 evolution and host adaptation, and they have potential implications for the development of effective control measures and vaccines, such as the design of targeted vaccines that account for regional variations in DHV-1 strains and the development of tailored control strategies that consider the interplay between virus evolution and host susceptibility.

## Figures and Tables

**Table 1:** Nucleotide contents and properties of the DHV-1 complete CDS.

Items	Ia	Ib	II	All
A	0.294 ± 0.001	0.291 ± 0.001	0.291 ± 0.001	0.292 ± 0.002
C	0.201 ± 0.001	0.197 ± 0.005	0.201 ± 0.003	0.2 ± 0.003
G	0.225 ± 0.001	0.227 ± 0.001	0.227 ± 0.001	0.226 ± 0.001
U	0.28 ± 0.001	0.285 ± 0.005	0.281 ± 0.003	0.281 ± 0.003
A3	0.269 ± 0.002	0.26 ± 0.003	0.264 ± 0.003	0.266 ± 0.004
C3	0.184 ± 0.003	0.174 ± 0.012	0.181 ± 0.008	0.182 ± 0.008
G3	0.2 ± 0.001	0.206 ± 0.003	0.203 ± 0.003	0.202 ± 0.003
U3	0.346 ± 0.002	0.36 ± 0.012	0.351 ± 0.009	0.35 ± 0.009
GC	0.426 ± 0.002	0.425 ± 0.006	0.427 ± 0.003	0.426 ± 0.003
AU	0.574 ± 0.002	0.575 ± 0.006	0.573 ± 0.003	0.574 ± 0.003
GC1	0.491 ± 0.001	0.492 ± 0.001	0.494 ± 0.003	0.492 ± 0.002
GC2	0.403 ± 0.001	0.402 ± 0.001	0.404 ± 0.001	0.403 ± 0.001
GC12	0.447 ± 0.001	0.447 ± 0.001	0.449 ± 0.001	0.448 ± 0.001
GC3	0.385 ± 0.004	0.38 ± 0.015	0.385 ± 0.01	0.384 ± 0.009
ENC	51.387 ± 0.233	50.595 ± 0.872	51.175 ± 0.712	51.184 ± 0.633

## Data Availability

The data supporting the findings of this study are available within the article.

## Supplementary Materials

The following supporting information can be downloaded at https://www.mdpi.com/article/10.3390/v16091380/s1, Figure S1: Recombination analysis of DHV-1 strains; Figure S2: PCA scree plot based on the RSCU values of the DHV-1 complete CDS; Figure S3: The variable correlation plots of PCA; Table S1: The nucleotide contents and properties of the DHV-1 complete CDS; Table S2: Synonymous codon usage of the DHV-1 complete CDS; Table S3: Relative dinucleotide abundance of the DHV-1 complete CDS.
