# Supplementary material for: Codon Usage Analysis Reveals Distinct Evolutionary Patterns and Host Adaptation Strategies in Duck Hepatitis Virus 1 (DHV-1) Phylogroups
Source: Viruses. 2024 Aug 29;16(9):1380. doi: 10.3390/v16091380 (PMC11437458; doi:10.3390/v16091380)
Supplement: Supplementary file 1 [file viruses-16-01380-s001.zip › Figures S1-S4.pdf]

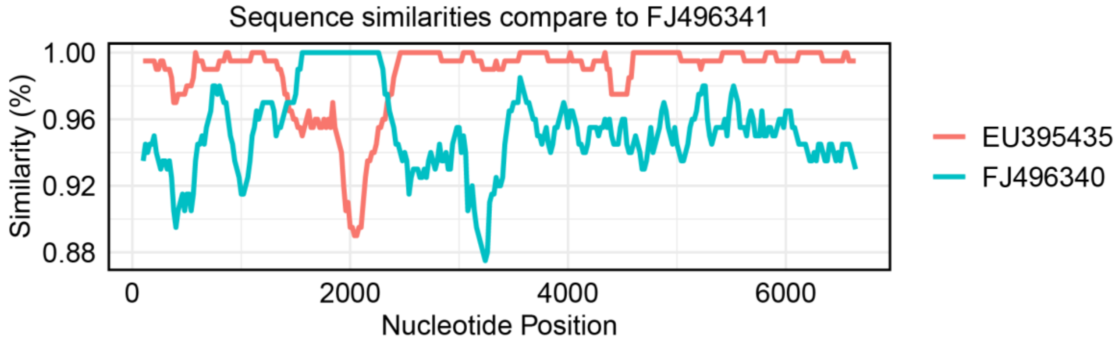

(a)

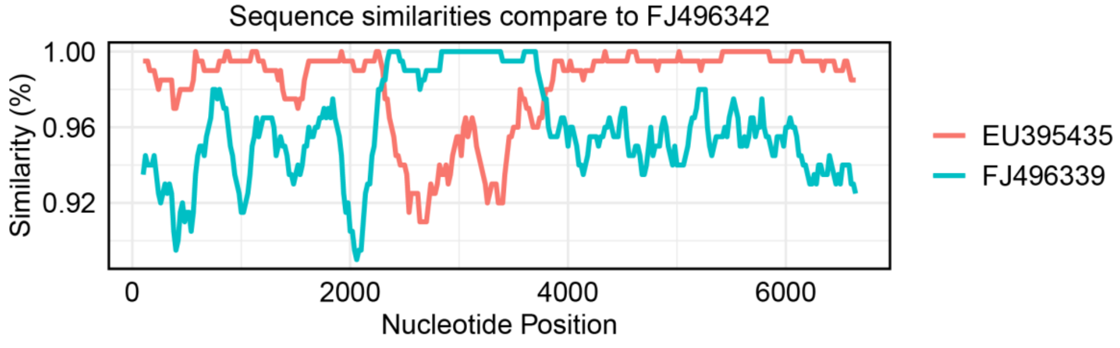

(b)

Figure S1. Recombination analysis of DHV-1 strains. (a) Recombination of the GFS06 (FJ496341) strain. The mosaic genomic structure of the DHV-1 GFS06 strain might originate from the JH2 (EU395435) strain and FFZ05 (FJ496340) strain, which belong to genotypes Ia and Ib, respectively. (b) Recombination of the GQY07 (FJ496342) strain. The GQY07 strain might be recombined from the JH2 (EU395435) strain and GHZ04 (FJ496339) strain, which belong to genotypes Ia and II, respectively.

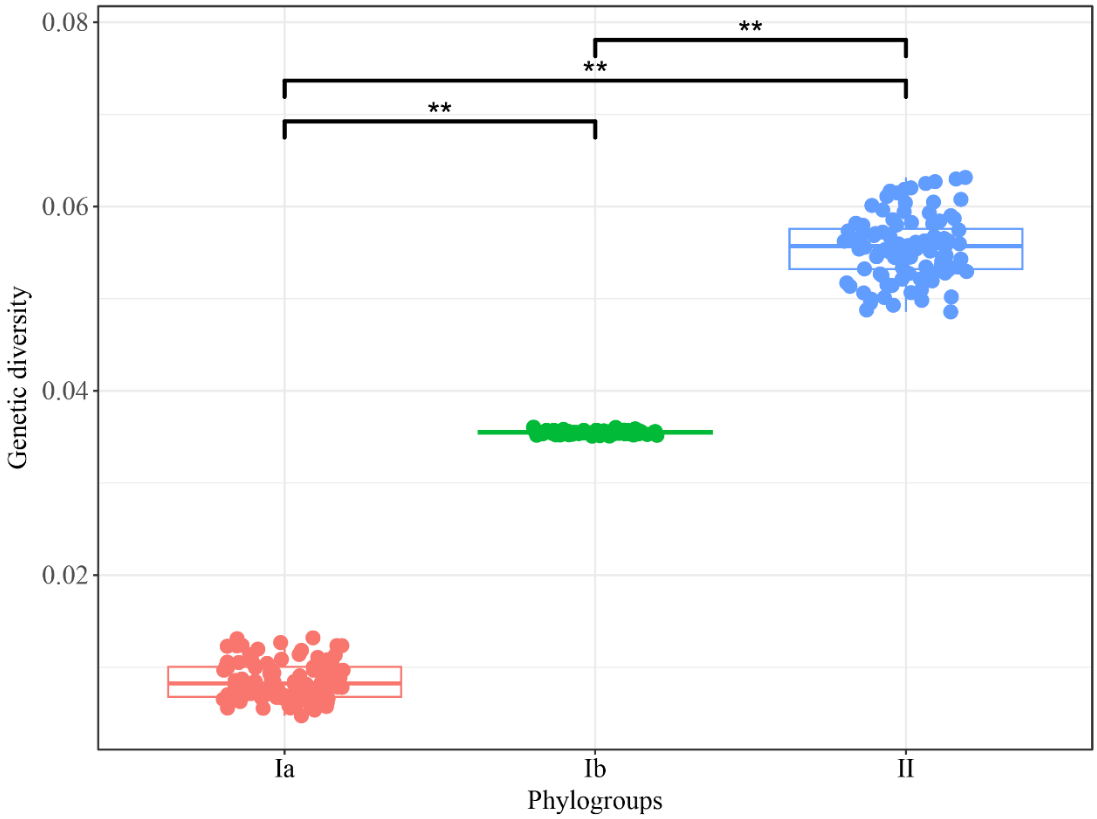

Figure S2. Subsampling analysis of genetic diversity. Double asterisks indicate significant differences at the 1% level. Phylogroups Ia, Ib, and II are represented in orange, green, and blue, respectively.

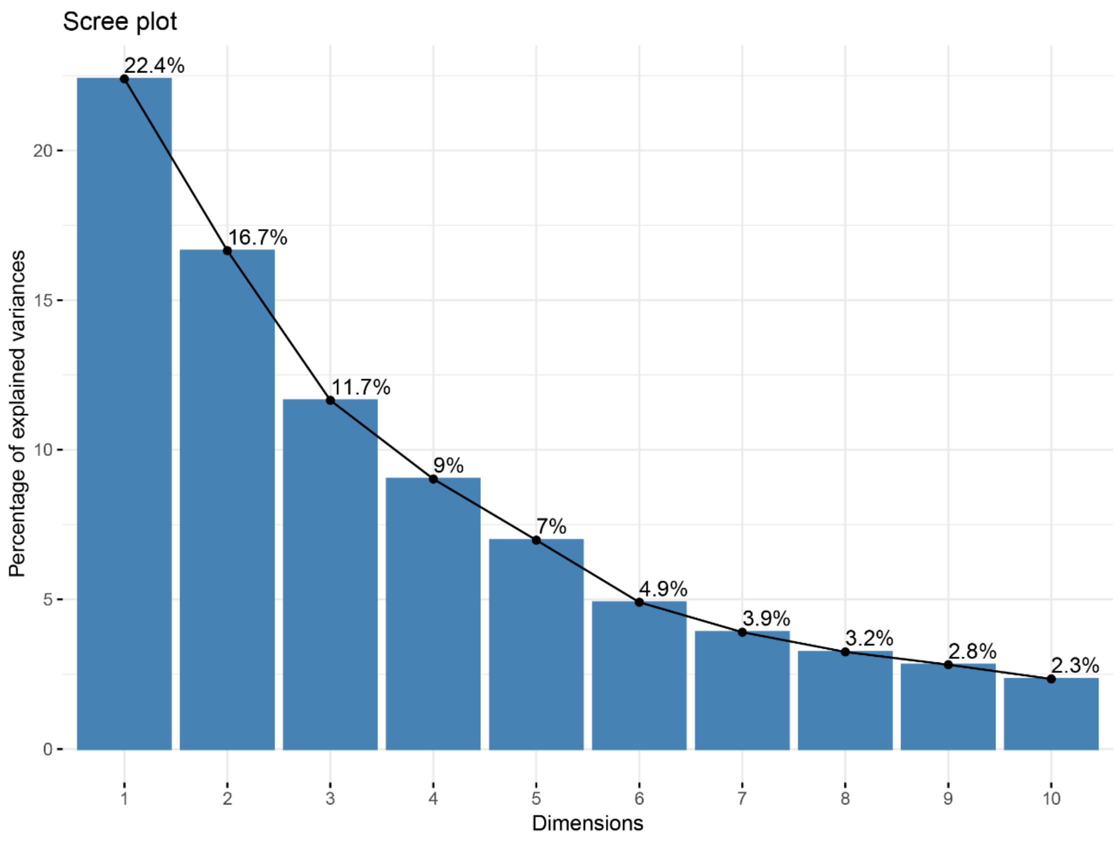

Figure S3. PCA scree plot based on the RSCU values of the DHV-1 complete CDS. The scree plot extracts and visualizes eigenvalues (variances) from the RSCU values and shows the proportion of total variance for each principal component (dimension) in descending order of magnitude.

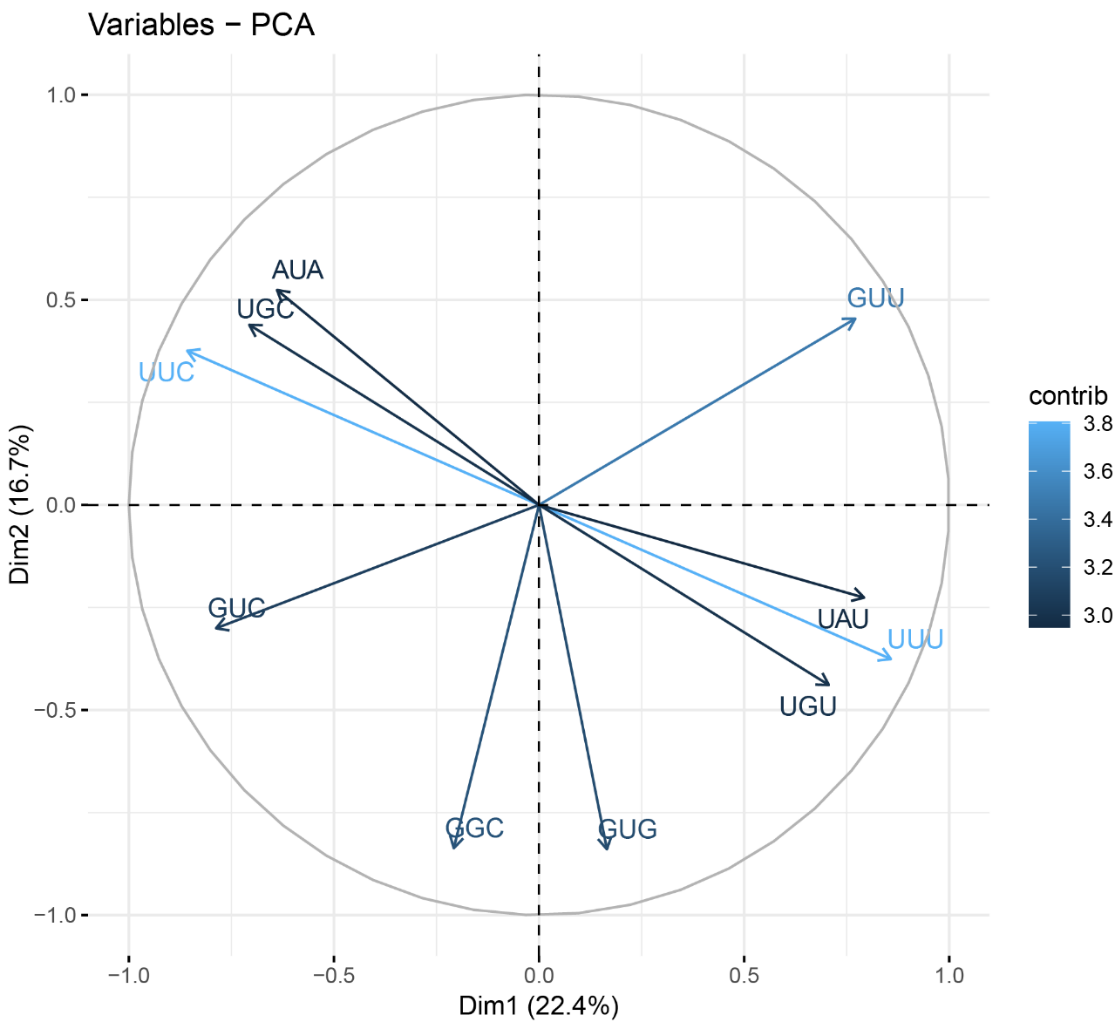

Figure S4. The variable correlation plots of PCA. The correlation circle with scaled coordinates of the variables' projections is represented. The top 10 variables and their directions are represented.
